# Pre‐Season Total Energy Expenditure and Dietary Intake of Professional Male Soccer Players: A Doubly Labelled Water Study

**DOI:** 10.1002/ejsc.70149

**Published:** 2026-02-27

**Authors:** Andrew Jenkinson, Ben Jones, Lucy Chesson, Lara Wilson, Rob Price, Catherine Hambly, John R. Speakman, Nessan Costello

**Affiliations:** ^1^ Carnegie School of Sport Leeds Beckett University Leeds UK; ^2^ Premiership Rugby London UK; ^3^ England Performance Unit Rugby Football League Leeds UK; ^4^ Faculty of Health Sciences School of Behavioural and Health Sciences Australian Catholic University Brisbane QLD Australia; ^5^ Division of Physiological Sciences and Health Through Physical Activity Department of Human Biology Faculty of Health Sciences Lifestyle and Sport Research Centre University of Cape Town Cape Town South Africa; ^6^ Scotland Rugby Union Murrayfield Stadium Edinburgh Scotland; ^7^ Leeds United Football Club Leeds UK; ^8^ Institute of Biological and Environmental Sciences University of Aberdeen Aberdeen UK; ^9^ State Key Laboratory of Molecular Developmental Biology Institute of Genetics and Developmental Biology Chinese Academy of Sciences Beijing China; ^10^ Shenzhen Key Laboratory of Metabolic Health Center for Energy Metabolism and Reproduction Shenzhen Institutes of Advanced Technology Chinese Academy of Sciences Shenzhen China

**Keywords:** nutrition, team sport, training

## Abstract

Limited data exist describing how professional footballers meet their energy requirements during pre‐season, a phase characterised by increased training volume and a progressive shift from general conditioning to football‐specific preparation. This study quantified total, resting, and activity energy expenditure (AEE), diet‐induced thermogenesis, water turnover, and dietary intake in six professional male soccer players (age: 25 ± 1 year; height: 182.5 ± 10.1 cm; body mass: 77.8 ± 8.2 kg). Players were studied across 14 consecutive days, representing training‐only and training‐plus‐match microcycles. Total energy expenditure (TEE) was measured using doubly labelled water, resting energy expenditure (REE) by indirect calorimetry and dietary intake using the remote food photography method. Fourteen‐day mean TEE, REE, AEE and water turnover were 13.25 ± 1.31 MJ⋅day^−1^, 7.96 ± 0.89 MJ⋅day^−1^, 4.20 ± 1.03 MJ⋅day^−1^, 5.16 ± 0.66 L⋅day^−1^, respectively. Physical activity level was 1.67 ± 0.16 AU. Energy, carbohydrate, protein, and fat intakes were 10.95 ± 1.52 MJ⋅day^−1^, 2.8 ± 0.6 g⋅kg^−1^⋅day^−1^, 2.2 ± 0.4 g⋅kg^−1^⋅day^−1^, and 1.5 ± 0.4 g⋅kg^−1^⋅day^−1^, respectively. Total energy expenditure was not significantly different between training‐only and training‐plus‐match microcycles (+1.89 ± 1.98 MJ⋅day^−1^; ES = 0.95 ± 1.08; *p* = 0.100). No significant differences were observed in energy or macronutrient intake across weekly microcycles (*p >* 0.068) or between days (*p* > 0.144). Players did not achieve energy balance or align dietary intake with day‐to‐day training demands, suggesting limited nutrition periodisation during pre‐season. These findings highlight the need for practitioners to implement strategies supporting fuelling, recovery and adaptation during this critical phase.

## Highlights

- Energy expenditure was similar between training‐only and training‐plus‐match microcycles, meaning players' fuelling requirements remained largely unchanged. Despite this, players still under‐consumed energy and carbohydrate, highlighting a consistent mismatch between demand and intake across pre‐season.
- Players did not achieve energy balance or meet carbohydrate recommendations, with no meaningful adjustment of intake across rest, single‐training, double‐training or match days, indicating limited nutrition periodisation during pre‐season.
- Meal‐by‐meal analysis showed low carbohydrate availability at key fuelling windows, particularly breakfast and post‐training lunch, while protein targets were met and fat intake exceeded recommendations. This underscores the need for targeted, periodised fuelling strategies.

## 1 Introduction

Soccer players require sufficient energy and nutrient intake to meet the demands of training and match‐play and to support overall health and well‐being (Mountjoy et al. 2018). Professional players typically complete 4–6 pitch‐based training sessions and 1–3 competitive matches per week during the in‐season period (Bangsbo et al. 2006; Anderson et al. 2022). Consequently, the average total daily energy expenditure (TEE) of professional players are ∼12.5–14.6 MJ⋅day^−1^ (∼3000–3500 kcal⋅day^−1^) (Brinkmans et al. 2019; Anderson et al. 2017a), corresponding to a physical activity level (PAL) of ∼1.75, or an active lifestyle (National Academies of Sciences E and M 2023). In preparation for in‐season, players typically complete ∼6 weeks of intensive pre‐season training, which aims to rebuild the physical fitness to meet the demands of competition (Jeong et al. 2011). Subsequently, the weekly training frequency, intensity and duration is increased (Anderson et al. 2022; Jeong et al. 2011) and players are exposed to 1–2 daily training sessions, 5–6 days per week (Jeong et al. 2011; Impellizzeri et al. 2006). Despite the differences in training demands between these distinct seasonal phases, there is a lack of research quantifying the TEE of senior male players during the pre‐season using criterion methods (Collins et al. 2021). Therefore, further investigation is warranted during this preparatory training phase.

Despite the role of nutrition in optimising physical performance, promoting training adaptation and supporting immune function (Thomas et al. 2016), simultaneous quantification of dietary intake and TEE in professional soccer players during a pre‐season is lacking. Soccer specific nutrition guidelines have historically encouraged high carbohydrate intakes, with previous recommendations advising 3–6 g·kg^−1^ body mass on training days and 6–8 g·kg^−1^ body mass on days requiring enhanced recovery and match preparation (Anderson et al. 2022; Collins et al. 2021). However, following the advancement in understanding of energy requirements and physical loading patterns of professional soccer players, as well as evidence to suggest enhanced skeletal muscle adaptations to aerobic (Impey et al. 2018) and high‐intensity interval type training (Morton et al. 2009), a periodised approach to carbohydrate intake that takes into account both day‐by‐day and meal‐by‐meal requirements has been suggested (Anderson et al. 2022). To date, no study has evaluated the dietary energy and macronutrient intakes of senior male players during pre‐season rest, training, and match days, alongside within‐day meal‐by‐meal distributions or energy availability (EA). This knowledge gap makes it challenging to determine appropriate energy intakes for players across the physically demanding pre‐season mesocycle, while practitioners are unclear whether dietary recommendations are actually being achieved at key eating opportunities within (e.g., pre‐, during‐, or post‐training or competition) or between (rest, single training, double training, or match) days.

Given the progressive increase in training intensity and the introduction of match play as pre‐season develops, players' energy expenditure and dietary intake would be expected to rise in parallel with external load. Match play imposes higher cardiovascular strain, greater muscle glycogen utilisation, and increased recovery energy demands compared with training sessions. Therefore, it was hypothesised that TEE would be greater during a microcycle including match play compared with a training‐only microcycle, and that dietary intake would not fully compensate for this elevated energy requirement. Therefore, the primary aim of this study was to quantify resting, activity and total energy expenditure, as well as water turnover, in senior male professional soccer players during the pre‐season period, across microcycles comprising training only or training plus match‐play, using criterion methods. The secondary aim was to evaluate dietary energy and macronutrient intake, meal‐by‐meal distribution, and energy availability, across single‐training, double‐training, match, and rest days.

## 2 Materials and Methods

### 2.1 Participants

Six male professional soccer players from the senior squad of an English Football League Championship team were purposefully recruited between 24th June 2019 and 27th June 2019 (1 participant from each positional group; goalkeeper, central defender, wide defender, central midfielder and forward). No starting wide midfielder volunteered to participate; therefore, a second central midfielder was recruited. Eligibility criteria included first team squad registration, > 18 years old, and being injury free at the start of the study. Participant characteristics are presented in Table 1. Participant 6 (wide defender) sustained an osteochondral injury to the talar dome of the right ankle on day 11 of the assessment period, resulting in immobilisation in an air cast boot for the remainder of the study. Following injury, the participant continued to provide DLW urine samples, dietary intake records, and ActiGraph based physical activity data, consistent with all other participants. GPS‐derived training and match load data were excluded due to the absence of outdoor training. Rehabilitation during the final three days included pool rehabilitation and an MRI scan on day 12, a gym‐based session targeting the upper body, core and cycling on day 13, and a second gym‐based session targeting lower body, core and pool work on day 14. Participants free from injury completed 88 ± 12% (range: 71%–100%) of total training duration. Match exposure was limited, three players (participants 2–4) each completed 45 min in Match 1, one player (participant 5) completed 45 min in match 2, and one player (participant 1) completed 25 min in match 1 and 93 min in match 3 (U23 match). Limited match exposure of this kind is typical in pre‐season due to planned squad rotation. Ethics approval was provided by the local ethics committee (66567). Participants provided written informed consent before participating in the study and consented to the sharing of individual data.

**TABLE 1:** Baseline participant characteristics.

Player (position)	Age (y)	Stature (cm)	Body Mass (kg)	Fat free Mass (kg)	Fat Mass (kg)	Body fat (%)	Professional league appearances (*n*)	International appearances (*n*)	International ranking	Highest league played
1 (CM)	19	165.0	62.2	54.3	8.4	13.4	0	0	N/A	U23 professional development league 2
2 (CD)	27	186.0	81.6	68.3	14.2	17.2	268	2	38	English premier league
3 (CM)	27	177.0	73.6	65.5	8.6	11.6	238	0	N/A	English premier league
4 (F)	25	189.0	82.4	74.1	9.5	11.4	223	0	N/A	English premier league
5 (GK)	22	193.0	85.2	69.5	16.6	19.3	48	7	35	English championship
6 (WD)	27	185.0	78.8	66.3	12.6	16.0	396	0	N/A	English championship
Mean ± SD	25 ± 1	182.5 ± 10.1	77.8 ± 8.2	69.4 ± 4.1	12.2 ± 2.4	14.9 ± 3.1	196 ± 147	2 ± 3	—	—

*Note:* Participant positions are shown in brackets.

Abbreviations: CD, central defender, CM, central midfielder; F, forward; GK, goalkeeper; WD, wide defender.

### 2.2 Study Design

Total, resting, and activity energy expenditure, diet‐induced thermogenesis, water turnover, dietary intake, training and match load, and physical activity were measured over 14‐day using a cross‐sectional research design (2019–2020 pre‐season). Microcycle 1 included training only (pre‐season microcycle), whereas microcycle 2 included training and match‐play (one‐match microcycle). Microcycle classification was determined by the team's planned schedule rather than individual match participation. Although individual match exposure varied, all players were exposed to the same overall training structure, recovery periods, and nutrition provision. All players followed the club's standardised pre‐season training programme focused on general conditioning and progressive football‐specific sessions, with no individualised training or body‐composition goals prescribed during this phase. On average, participants completed 5 ± 1 single training days, 5 ± 1 double training days, 1 ± 1 match days, and 3 ± 1 rest days across the assessment period. To enable sufficient match exposure for participants not involved in match 1, two additional matches were scheduled on days 13 and 14. Consequently, participant 5 completed 45 min in match 2 (day 13) and participant 1 completed 93 min in match 3 (day 14).

**FIGURE 1:**
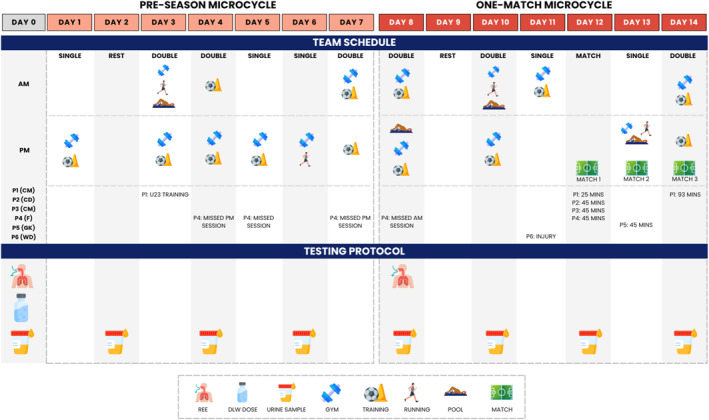
An overview of the schedule for the fourteen‐day assessment period. Participant positions are shown in brackets. CD, central defender; CM, central midfielder; DLW, doubly labelled water; F, forward; GK, goalkeeper; REE, resting energy expenditure; WD, wide defender.

Resting metabolic rate (RMR) was measured using indirect calorimetry. Activity energy expenditure (AEE) and TEE were measured by doubly labelled water (DLW). Dietary intake and distribution were assessed using a version of the remote food photographic method (RFPM). Internal and external load were measured using sessional ratings of perceived exertion (sRPE) and global positioning system (GPS) technology, respectively. Daily physical activity (outside of training and match‐play) was measured using a wrist‐worn triaxial accelerometer. Body composition and water turnover were measured by deuterium isotope dilution. The 14‐day schedule is presented in Figure 1.

### 2.3 Baseline Measures

On the morning of day 0, fasted body mass (BM) (SECA, model‐875, Hamburg, Germany) and stature (SECA, model‐213, Hamburg, Germany) were measured to the nearest 0.2 kg and 0.1 cm, respectively, according to the International Society for the Advancement of Kinanthropometry guidelines. Fasted BM was also collected daily, excluding rest days.

### 2.4 Measurement of Total Energy Expenditure Using the Doubly Labelled Water Method

Total energy expenditure was measured using the DLW method (Lifson and McClintock 1966). Doubly labelled water has previously been validated in humans against simultaneous indirect calorimetry on multiple occasions (Speakman 1997), reporting excellent accuracy at a group level (average error: 0.64 ± 12.2%) (Speakman et al. 2021).

Doses of DLW were prepared in advance according to the individual BM of each participant, with a desired enrichment of 10 atom percent excess ^18^O and 5 atom percent excess deuterium, using the calculation:

(1) dose(mL)=0.65(bodymass,g)xDIE/IE

where 0.65 is the approximate proportion of the body comprising water, DIE is the desired initial enrichment (DIE = 618.923 × body mass, kg^−0.305^) and IE is the initial enrichment (10%) 100,000 parts per million (Speakman 1997).

On day 0, participants provided a baseline urine sample (second pass of the day) and then consumed a single dose of DLW (08:00–10:00). A second urine sample was collected after 9 ± 0 h to determine initial isotope enrichment following total body water equilibrium (Speakman 1997). Participants engaged in representative activities during the equilibrium period (*ad libitum* food and drink consumption and typical training including morning aerobic interval running, upper body resistance exercise, pool‐based activity, and afternoon field‐based training).

Urine samples were collected into 25 mL containers (Sarstedt AG & Co. KG, Nümbrecht, Germany) every other morning (second pass of the day) and recorded to the nearest minute by the principal researcher until day 15. Urine samples were temporarily stored in a portable fridge and later transported and stored at −80°C in 2 mL micro tubes (Sarstedt AG & Co. KG, Nümbrecht, Germany) until later analysis (Westerterp 2017).

Analysis of the isotopic enrichment of urine was performed blind using a Liquid Isotope Water Analyser (Los Gatos research, United States of America) (Berman et al. 2012). Initially the urine was encapsulated in capillaries, then vacuum distilled (Nagy 1983), and the resulting distillate used for analysis. Samples were run alongside five laboratory standards for each isotope and international standards to adjust for day‐to‐day variation and allow correction from delta values to parts per million. After adjustment for background levels, daily isotope enrichments were log converted and the elimination constants (*k*_*o*_ and *k*_*d*_) were calculated by fitting a least squares regression model to the log converted data. The back extrapolated intercept was used to calculate the isotope dilution spaces (*N*_*o*_ and *N*_*d*_) by multi‐point analysis (Microsoft Excel, version 16.65, 2022). A two‐pool method, specifically Equation (1) from Speakman and colleagues (Speakman et al. 2021), was used to calculate rates of CO_2_ production.

(2) $$\text{rCO}2=\left[\left(N/2.078\right)\;*\;\left(1.007\;*\;k_{o}-1.043\;*\;k_{d}\right)-\left(0.0246\;*\;N\;*\;1.05\left(1.007\;*\;k_{o}-1.043\;*\;k_{d}\right)\right)\right]*\;22.26$$

where N is total body water. From this, rCO_2_ was converted to TEE, using the Weir equation and an estimated respiratory quotient of 0.85 for all participants (*equation 5* from Speakman and colleagues) (Speakman et al. 2021).

(3) $$\text{TEE}\;\left(\text{MJ}/d\right)=\text{rCO}2\;*\;\left(1.106+\left(3.94/\text{RQ}\right)\right)\;*\;\left(4.184/10^{3}\right)$$

The results have been reported as a single 14‐day average, alongside two 7‐day averages (pre‐season microcycle and one‐match microcycle). Physical activity level (PAL) was calculated by dividing TEE by measured RMR. The thermic effect of food (TEF) was assumed to be 10% of EI for each participant (Westerterp 2004), subsequently enabling estimations of activity energy expenditure (AEE; TEE—(RMR + TEF)) and energy availability (EA = EI—AEE/FFM) (Loucks 2004). Energy availability was calculated using AEE derived from DLW, which encompasses both EEE and non‐exercise activity thermogenesis (NEAT). This method is consistent with the ‘improved EA’ approach proposed by Taguchi et al. (2022) for free‐living athletes and has been applied in recent DLW investigations of professional male and football players (Hannon et al. 2021; Morehen et al. 2022).

### 2.5 Body Composition and Water Turnover

Body composition and water turnover was measured by deuterium isotope dilution. Total body water was calculated from the stable isotope dilution spaces based on the intercept of the deuterium elimination plot (International Atomic Energy Agency 2009):

(4) $$N=[(N_{o}/1.007)+(N_{d}/1.043]/2$$

whereby, *N*_*o*_ and *N*_*d*_ are the ^18^O and deuterium dilution space, respectively (Speakman et al. 2021).

Fat‐free mass (FFM) (kg) was determined using a two‐compartmental model of body composition, by dividing total body water (kg) by 73.2 (Widdowson et al. 1951). Fat mass (kg) was calculated by subtracting FFM from initial BM (kg).

Water turnover was calculated by multiplying the rate constant of the post‐dose decline in deuterium enrichment by the total water pool (Yamada et al. 2022).

### 2.6 Assessment of Resting Metabolic Rate

Resting metabolic rate was measured on day 0 and day 8 by open‐circuit indirect calorimetry (Cortex 3B‐R3 MetaLyzer, CORTEX Biophysik GmbH, Leipzig, Germany), under standardised conditions (i.e., overnight fast, > 8‐h abstention from alcohol, nicotine, and caffeine) (Fullmer et al. 2015) at the club training ground (08:00–10:00). Prior to each assessment, the calorimeter was calibrated to within 0.02% of two known gas concentrations (15% O_2_ and 5% CO_2_), and ambient air (20.93% and 0.04%). A Hans Rudolph silicon face masque (7450 Series V2; Hans Rudolph Inc., Kansas, MO) was positioned over the participants nose and mouth while lying in a comfortable supine position, within a quiet, dimly lit and thermoneutral (20°C–22°C) room (Compher et al. 2006).

Data were collected over a twenty‐minute period, following 5 minutes of rest. Ventilatory O_2_ ($\dot{V}$O_2_) and CO_2_ ($\dot{V}$CO_2_) were measured continuously and averaged every 30 s to remove artefacts. After discarding the first 5‐min of data collection, the five‐minute period with the lowest coefficient of variation (CV) for $\dot{V}$O_2_, $\dot{V}$CO_2_ and respiratory exchange ratio (RER) was chosen for analysis that met the steady state criteria (i.e., CV < 10% for $\dot{V}$O_2_ and $\dot{V}$CO_2_, alongside CV < 5% for RER) (Fullmer et al. 2015). One participant presented with a CV > 10% and > 5% for $\dot{V}$O_2_ and $\dot{V}$CO_2_ and RER, respectively (final group CV for $\dot{V}$O_2_: 8.2 ± 3.9%; $\dot{V}$CO_2_: 9.3 ± 5.0%; RER: 4.3 ± 3.4%). Data were exported to Microsoft Excel, for the calculation of substrate oxidation rates by the Weir equation (Weir 1949). Energy expenditure was estimated using an energy value for carbohydrate and fat of 3.75 and 9 kcal per gram, respectively (Southgate and Durnin 1970). Resting metabolic rate measured on day 8 are solely presented, due to error with calibration gas values on day 0 that influenced measured RMR values.

### 2.7 Assessment of Energy and Macronutrient Intake

Self‐reported energy and macronutrient intake was measured using a version of the RFPM (Martin et al. 2008), which has previously been validated in adolescent athletes (Costello et al. 2017) and used with senior professional soccer players (Anderson et al. 2017a; Capling et al. 2017). The participants provided one before and after photograph of their food and drink consumption, alongside a written or audio recorded description of each item (quantity, brand, and ingredients) over a free encrypted picture messaging smartphone application (WhatsApp Inc., California, USA). Where any items were not consumed in full, a second image and description were provided. The lead researcher was present at all club provided eating opportunities to support accurate reporting (training ground, travel, pre‐, during‐ and post‐match meals). Additionally, the lead researcher weighed and photographed standard portion sizes of individual food or drink items, creating a picture‐based database to assist with retrospective portion size estimation for each eating occasion at the club training ground. Menus and recipes were also provided to the club catering team as part of standard practise, which further supported retrospective dietary analysis.

Energy and macronutrient intakes were measured by a Sport and Exercise Nutrition register (SENr) accredited nutritionist, using professional dietary analysis software (Nutritics Ltd., version 5.096, Dublin, Ireland). To ensure reliability of the analysed intakes, two additional SENr accredited practitioners independently assessed all dietary intakes (84 days in total). Reported energy and macronutrient intakes are the average of the three independent dietary analysis, to account for the error typically reported by researchers during dietary analysis (Braakhuis et al. 2003; Stables et al. 2021). The inter‐researcher CV for all players over the 14‐day was: energy: 4 ± 0%; CHO: 3 ± 0%; protein: 4 ± 0%; fat: 3 ± 0%; and alcohol: 0 ± 0%. The inter‐researcher CV for energy and macronutrient intakes at each eating occasion are displayed in Supporting Information S1: Figure 1.

### 2.8 Quantification of Internal and External Load

Internal load was measured using sRPE. Thirty minutes following the end of each field‐based training session, participants individually reported their rating of perceived exertion to the lead sports scientist, using a modified Bourg scale, which was multiplied by session duration to calculate load in arbitrary units (AU) (Foster et al. 2001).

External pitch‐based training and match loads were quantified using a GPS micro‐technology unit (OptimEye S5, Catapult Innovations, Scoresby, Victoria) which included 10 Hz GPS, 100‐Hz triaxial accelerometer, gyroscope, and magnetometer. The unit was positioned between the scapulae within a manufacturer designed vest and operated by the lead sports scientist according to typical procedures (Malone et al. 2017). A 10 Hz GPS has been reported to be valid and reliable for quantifying distance and speed measurements during team sport activities (Malone et al. 2017). The GPS units were activated outside ∼30 min prior to use to obtain a satellite lock. Following the completion of each session, data was downloaded using manufacturer's software (Openfield, version 2.5.0, Catapult Sports, Melbourne, Australia). The external load variables selected for analysis were total duration (in minutes), total distance covered (in metres), high speed running (HSR) (19.8–25.1 km·h^−1^) and sprinting (> 25.1 km·h^−1^). Participant 5 (goalkeeper) did not wear a GPS unit throughout the study. As a result, external load data (e.g., total distance, high‐speed running) were unavailable for Participant 5, and this participant was therefore excluded from the training and match‐load analyses only.

### 2.9 Assessment of Physical Activity

Physical activity was measured using a research‐grade accelerometer (tri‐axial ActiGraph GT9‐X Link accelerometer, Pensacola, Florida, USA). The device was worn continuously on the wrist, except during water‐based activities, field‐based training, and match‐play. Participants wore the accelerometer for an average of 98 ± 2% of the 14‐day assessment period (range: 90%–100%). The devices were initialised using participant information (ActiLife software, version 6.13.3: ActiGraph) and data were collected continuously with a sampling frequency of 30 Hz. Data were downloaded and wear time validation performed during field‐based training on day 8 and day 15 for the pre‐season and one‐match microcycle, respectively. Valid days had a minimum of 10 h of wear time (non‐wear time was defined as 90 consecutive minutes of zero counts (Choi et al. 2012)) and three valid days was required for analysis (all participant days were included for analysis). Sedentary (< 2000 vector magnitude (VM) counts/min), light (2000–7999 VM counts/min), and moderate‐to‐vigorous physical activity (> 8000 VM counts/min) was defined using specific cut points (Mikkelsen et al. 2020; Reid et al. 2017). Total step count was calculated using ActiLife proprietary software. Accelerometer‐derived data (step count, sedentary time, and light activity expressed as a percentage of daily wear time) were analysed and are presented in the Results to provide contextual information on physical activity undertaken outside of training and match‐play. Individual accelerometer‐derived physical activity data across weekly microcycles are presented in Supporting Information S1: Table 4.

### 2.10 Statistical Analysis

SPSS (version 28; SPSS, Chicago, IL) was used for statistical analysis. Normality of distribution for all continuous variables was assessed using the Shapiro–Wilk test. All variables met normality assumptions. Participant 6 has been excluded from all statistical analysis due to injury. Participant 5 (goalkeeper) was excluded from the training and match‐load analyses only, as no GPS data were collected for this position in line with standard club practise. Participant 5 was retained in all other analyses, including energy expenditure, dietary intake, and physical activity. Paired *t‐*tests were used to compare differences between microcycles (pre‐season and one‐match). Differences in energy and macronutrient intake between days (single and double training, match, and rest) and relative energy and macronutrient distribution between eating occasions (before breakfast vs. breakfast vs. morning snack vs. lunch vs. afternoon snack vs. dinner vs. evening snack) were assessed using one‐way repeated‐measures ANOVA. Assumptions of sphericity were tested using Mauchly's test, and where violations were detected, Greenhouse‐Geisser corrections were applied, otherwise, sphericity was assumed. Where significant main effects were present, Tukey *Post hoc* analysis was conducted. Statistical significance was set at *p* < 0.05. Data are presented as mean ± SD. To determine the magnitude of the difference, effect size (ES) according to Cohen *d* (Cohen 2013) procedure was calculated.

## 3 Results

### 3.1 Baseline Characteristics

Participants (*n* = 6) were senior male professional soccer players aged 25 ± 1 year, with a mean stature of 182.5 ± 10.1 cm, body mass of 77.8 ± 8.2 kg, and body‐fat percentage of 14.9 ± 3.1%. Mean fat‐free mass was 69.4 ± 4.1 kg. Participant characteristics are presented in Table 1.

### 3.2 Quantification of Training Load, Match Load and Physical Activity

#### 3.2.1 Pre‐Season versus One‐Match Microcycle

**FIGURE 2:**
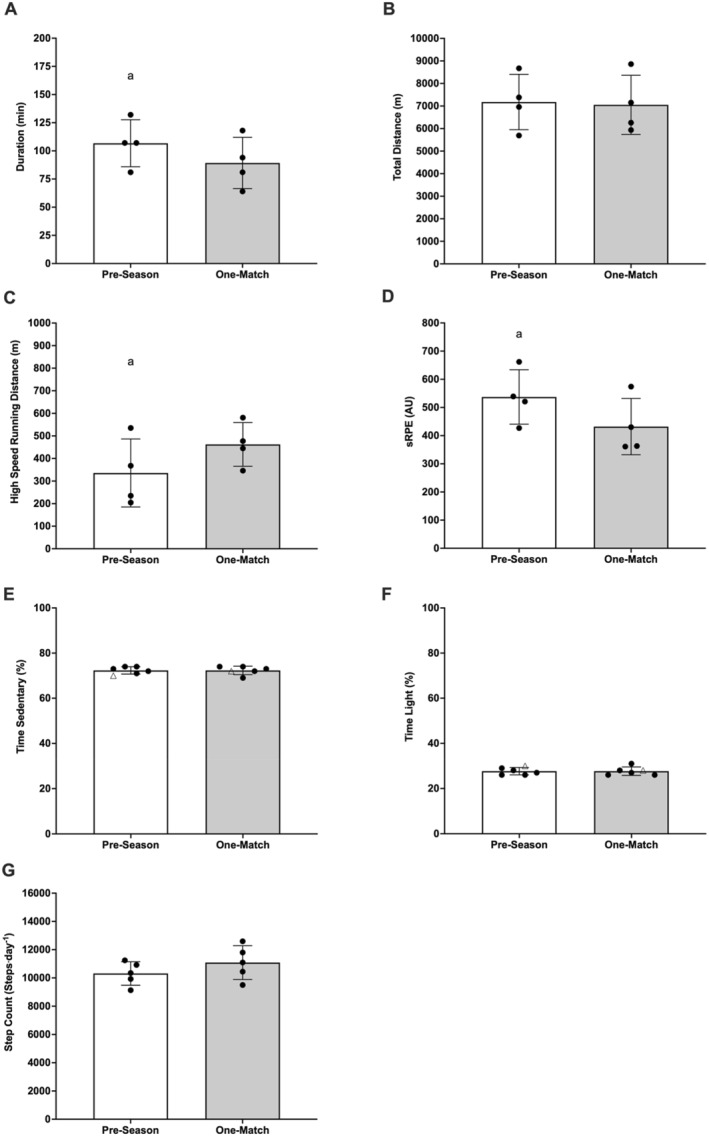
Training and match duration (A), total distance (B), high‐speed running (C), sessional rating of perceived exertion (D), time in sedentary activity (E), time in light activity (F) and step count (G) across weeks. White bars represent a pre‐season microcycle. Light grey bars represent a one‐match microcycle. ^a^Significant difference from one‐match microcycle, *p* < 0.05. Black circles represent individual outfield participants. White triangle represents participant 5 (goalkeeper). Data not included for participant 6 (injured). Sessional rating of perceived exertion (sRPE). Arbitrary units (AU).

Session duration (Figure 2A) (18 ± 6 min; ES = 2.96 ± 2.44; *p* = 0.010) and sRPE (Figure 2D) (105 ± 50 AU; ES = 2.11 ± 1.87; *p* = 0.024) were significantly greater during the pre‐season than one‐match microcycle. High‐speed running (Figure 2C) was significantly lower (−127 ± 68 m; ES = −1.85 ± 1.71; *p* = 0.034) during the pre‐season than one‐match microcycle. All other differences in load (*p* > 0.607) and physical activity (*p* > 0.129) were non‐significant across microcycles. Data are presented in Figure 2A–G. Individual player GPS‐derived external and internal load data across weekly microcycles are presented in Supporting Information S1: Table 1.

#### 3.2.2 Single Training versus Double Training versus Match versus Rest Days

**FIGURE 3:**
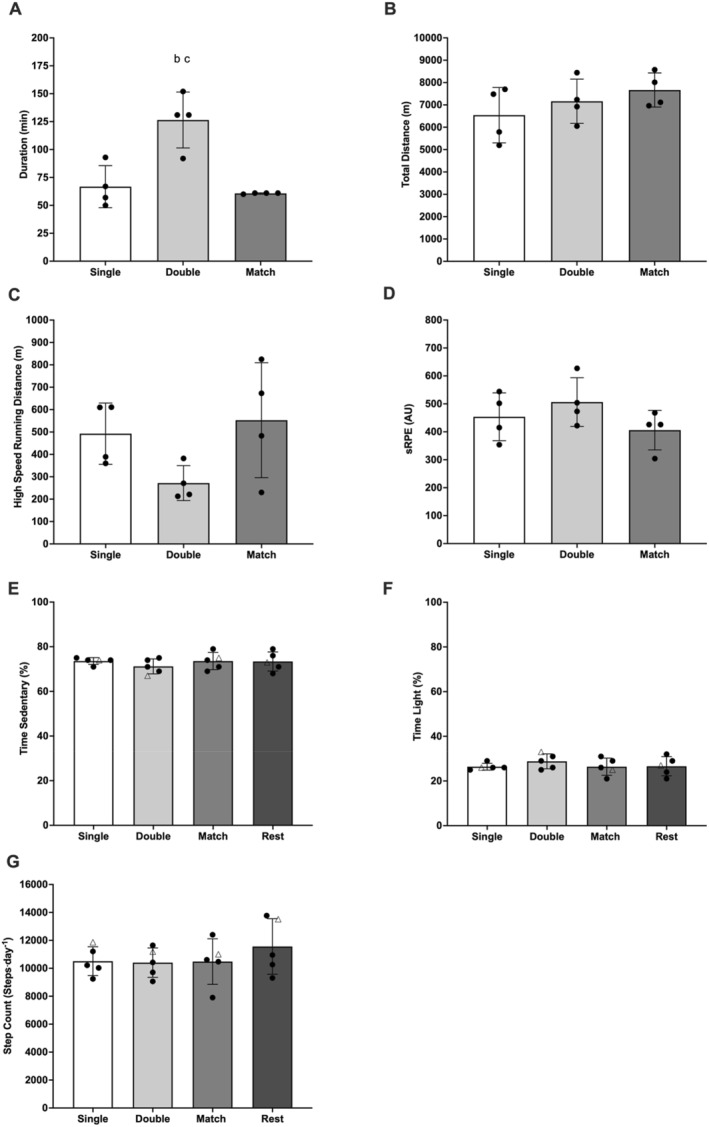
Training and match duration (A), total distance (B), high‐speed running (C), sessional rating of perceived exertion (D), time in sedentary (E), time in light activity (F) and step count (G) across days. White bars represent single training sessions. Light grey bars represent double training sessions. Grey bars represent match days. Dark grey bars represent rest days including no training sessions. Black circles represent individual outfield participants. White triangle represents participant 5 (goalkeeper). Data not included for participant 6 (injured). ^b^Significant difference from single training session, *p* < 0.05. ^c^Significant difference from match, *p* < 0.05.

Session duration (Figure 3A) was significantly greater on double training days than single training (60 ± 7 min; ES 4.47 ± 3.49; *p* = 0.009) and match (66 ± 13 min; ES 2.59 ± 2.19; *p* = 0.042) days. All other differences in load (*p* > 0.108) and physical activity (*p* > 0.506) were non‐significant across days. Data are presented in Figure 3A–G.

#### 3.2.3 Energy Expenditure and Water Turnover

Mean TEE and WT for the non‐injured cohort was 13.25 ± 1.31 MJ⋅day^−1^ and 5.16 ± 0.66 L⋅day^−1^ over the 14‐day period, respectively. Baseline RMR was 7.96 ± 0.89 MJ⋅day^−1^. Therefore, AEE was 4.20 ± 1.03 MJ⋅day^−1^ and PAL was 1.67 ± 0.16 AU.

The TEE and WT of the injured participant was 12.77 MJ⋅day^−1^ and 5.4 L⋅day^−1^ across the 14‐day period, respectively. Baseline RMR was 8.68 MJ⋅day^−1^. Therefore, AEE was 3.13 MJ⋅day^−1^ and PAL was 1.47 AU.

#### 3.2.4 Pre‐Season Versus One‐Match Microcycle

**FIGURE 4:**
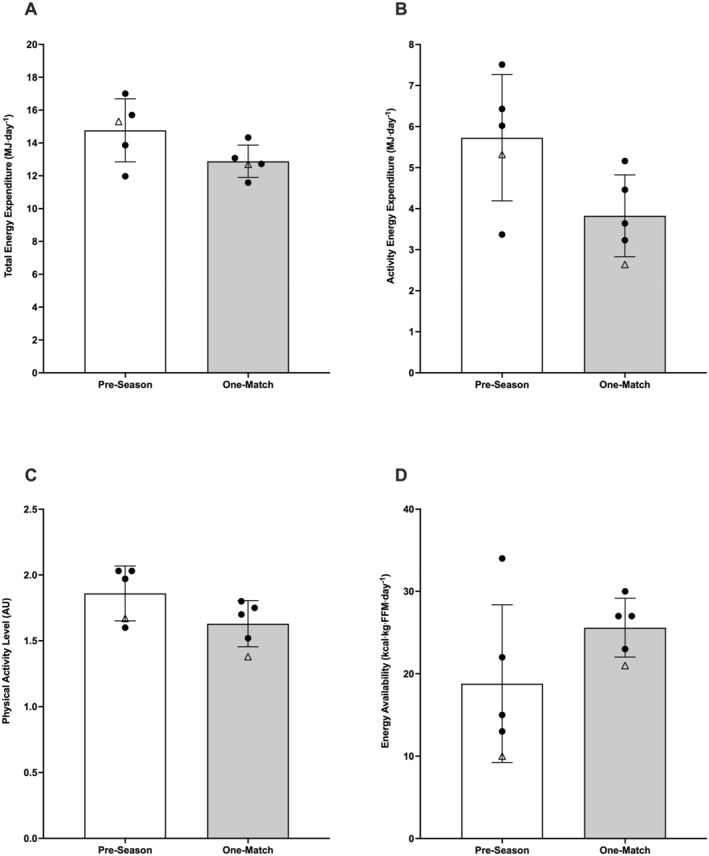
Total energy expenditure (A), activity energy expenditure (B), physical activity level (C) and energy availability (D). The white bars represent the pre‐season microcycle. The light grey bars represent the one‐match microcycle. Black circles represent individual outfield participants. White triangle represents participant 5 (goalkeeper). Values were removed for participant 6 (injured). Arbitrary units (AU).

There was no significant difference in TEE (1.89 ± 1.98 MJ⋅day^−1^; ES = 0.95 ± 1.08; *p* = 0.100), AEE (1.90 ± 1.99 MJ⋅day^−1^; ES = 0.95 ± 1.08; *p* = 0.099), PAL (0.23 ± 0.24 AU; ES 0.96 ± 1.09; *p* = 0.098), or water turnover (0.40 ± 0.36 L⋅day^−1^; ES = 1.11 ± 1.15; *p* = 0.068) across the pre‐season and one‐match microcycle. Data are presented in Figure 4A–C. Individual TEE, REE, AEE, diet‐induced thermogenesis (DIT), and WT data across weekly microcycles are presented in Supporting Information S1: Table 3.

Total energy expenditure, AEE, PAL, and water turnover for the injured participant during microcycle 1 was 15.70 MJ⋅day^−1^, 6.09 MJ⋅day^−1^, 1.81 AU, and 5.6 L⋅day^−1^ respectively. Following injury, TEE, AEE, PAL, and WT during microcycle 2 was 10.39 MJ⋅day^−1^, 0.72 MJ⋅day^−1^, 1.20 AU, and 5.1 L⋅day^−1^.

#### 3.2.5 Self‐Reported Energy and Macronutrient Intake

All relative intakes are expressed per kilogramme of body mass (g·kg^−1^ day^−1^ or MJ·kg^−1^ day^−1^). Mean energy intake for the non‐injured cohort was 10.95 ± 1.52 MJ⋅day^−1^ over the 14‐day period. Mean carbohydrate, protein, and fat intake was 211 ± 29 g⋅day^−1^ (2.8 ± 0.6 g⋅kg^−1^⋅day^−1^), 167 ± 10 g⋅day^−1^ (2.2 ± 0.4 g⋅kg^−1^⋅day^−1^), and 119 ± 23 g⋅day^−1^ (1.5 ± 0.4 g⋅kg^−1^⋅day^−1^), respectively. On average, carbohydrate, protein, and fat contributed 33 ± 4%, 26 ± 2%, and 41 ± 8% of total energy intake, respectively.

Mean energy intake for the injured participant was 9.67 ± 2.37 MJ⋅day^−1^ over the 14‐day period. Mean carbohydrate, protein, and fat intake was 197 ± 67 g⋅day^−1^ (2.4 ± 0.8 g⋅kg^−1^⋅day^−1^), 151 ± 47 g⋅day^−1^ (1.8 ± 0.6 g⋅kg^−1^⋅day^−1^), and 96 ± 26 g⋅day^−1^ (1.2 ± 0.3 g⋅kg^−1^⋅day^−1^), respectively. On average, carbohydrate, protein, and fat contributed 34 ± 6%, 26 ± 6%, and 37 ± 6% of total energy intake, respectively.

#### 3.2.6 Pre‐Season Versus One‐Match Microcycle

**FIGURE 5:**
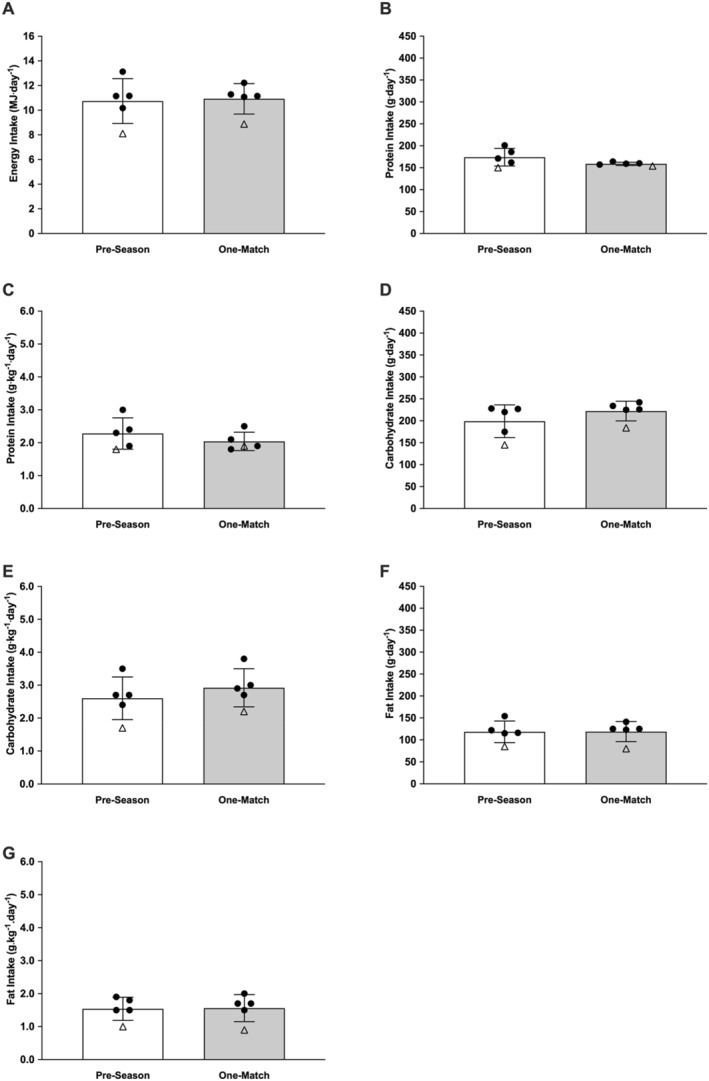
Energy intake (A), protein intake (B), relative protein intake (C), carbohydrate intake (D), relative carbohydrate intake (E), fat intake (F) and relative fat intake (G) across microcycles. The white bars represent the pre‐season microcycle. The light grey bars represent the one‐match microcycle. Black circles represent individual outfield participants. White triangle represents participant 5 (goalkeeper). Values were removed for participant 6 (injured).

There was no significant difference in energy (*p* = 0.661) or macronutrient (*p* = 0.068) intake across the pre‐season and one‐match microcycle. Data are presented in Figure 5A–G. Individual dietary intake data across weekly microcycles are presented in Supporting Information S1: Table 2.

#### 3.2.7 Single Training Versus Double Training Versus Match versus Rest Days

**FIGURE 6:**
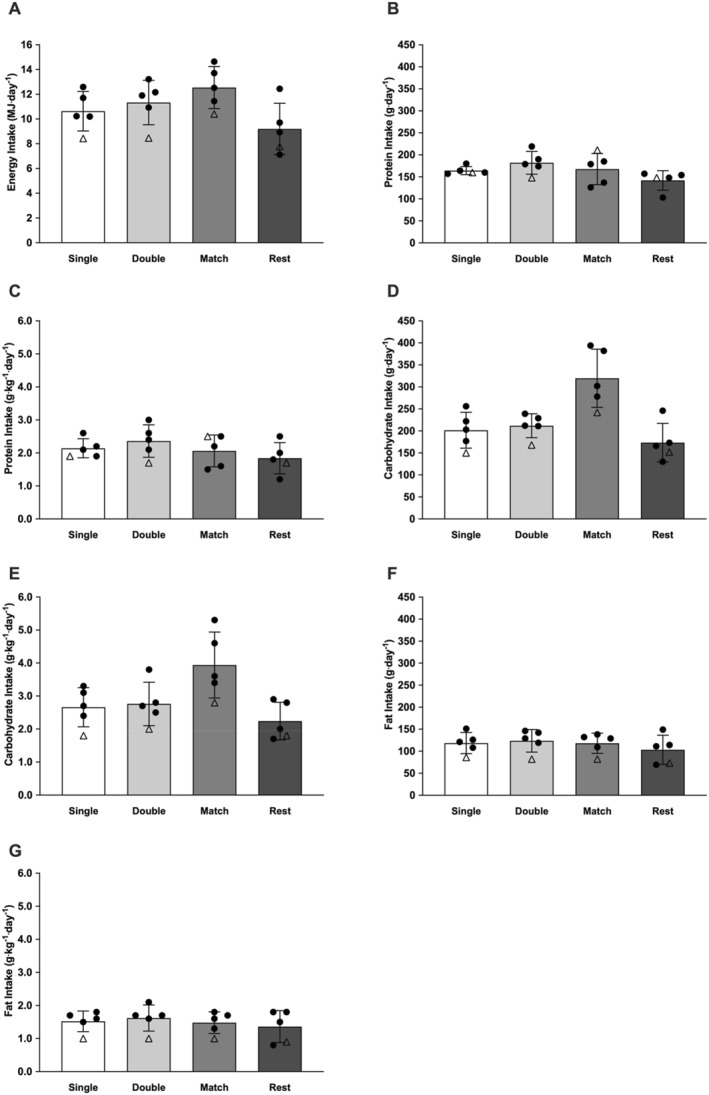
Energy intake (A), protein intake (B), relative protein intake (C), carbohydrate intake (D) relative carbohydrate intake (E), fat intake (F) and relative fat intake (G) across days. White bars represent training days including one session. Light grey bars represent training days including two sessions. Grey bars represent match days. Dark grey bars represent rest days including no training sessions. Black circles represent individual outfield participants. White triangle represents participant 5 (goalkeeper). Values were removed for participant 6 (injured).

All differences were non‐significant in absolute or relative energy (*p* > 0.641) or macronutrient (*p* > 0.144) intake across single training, double training, match, and rest days. Data is presented in Figure 6A–G.

### 3.3 Energy and Macronutrient Distribution

**FIGURE 7:**
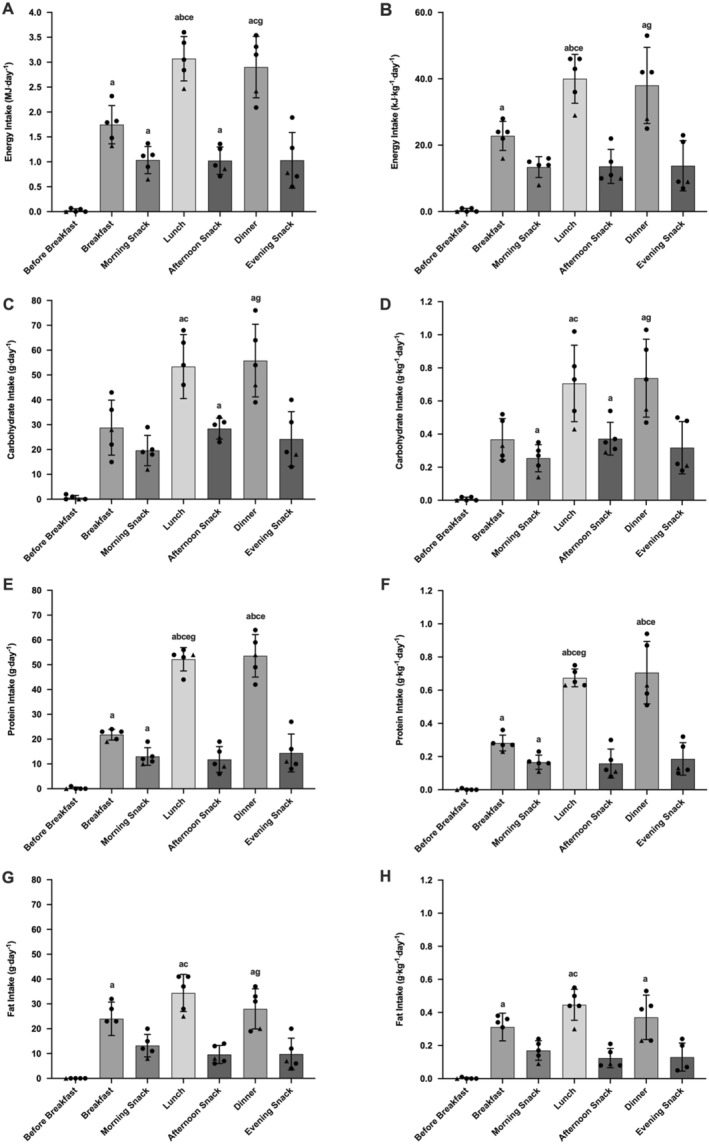
Energy and macronutrient distribution across meals. Absolute energy intake (A), relative energy intake (B), absolute carbohydrate intake (C), relative carbohydrate intake (D), absolute protein intake (E), relative protein intake (F), absolute fat intake (G) and relative fat intake (H). ^a^Significant difference from before breakfast, *p* < 0.05. ^b^Significant difference from breakfast, *p* < 0.05. ^c^Significant difference from morning snack, *p* < 0.05. ^d^Significant difference from lunch, *p* < 0.05. ^e^Significant difference from afternoon snack, *p* < 0.05. ^f^Significant difference from dinner, *p* < 0.05. ^g^Significant difference from evening snack, *p* < 0.05. Black circles represent individual outfield participants. White triangle represents participant 5 (goalkeeper). Values were removed for participant 6 (injured).

Over the 14‐day period relative energy intake was significantly greater at lunch than breakfast (*p* = 0.012). Relative carbohydrate intake was significantly greater at afternoon snack than before breakfast (*p* = 0.011), and at morning snack than before breakfast (*p* = 0.036). Relative protein intake was significantly greater at dinner (*p* = 0.042) and lunch (*p* = 0.004) than breakfast. Relative protein intake was significantly greater during morning snack than before breakfast (*p* = 0.046). All other differences between meals (breakfast vs. lunch vs. dinner) and snacks (before breakfast vs. morning snack vs. afternoon snack vs. evening snack) were non‐significant (*p* > 0.054). Energy and macronutrient distribution data is presented in Figure 7A–H.

### 3.4 Energy Balance and Availability

Mean energy intake for the non‐injured cohort was significantly lower than TEE during the pre‐season (−4.01 ± 2.56 MJ⋅day^−1^; ES = −1.57 ± 1.36; *p* = 0.025) and one‐match (−1.96 ± 1.21 MJ⋅day^−1^; ES = −1.62 ± −1.39; *p* = 0.022) microcycle. There was no significant difference in BM across the 14‐day period (0.10 ± 0.24 kg; ES = 0.41 ± 0.92; *p* = 0.413).

Mean energy availability for the non‐injured cohort was 24.8 ± 6.3 kcal·kg^−1^·FFM^−1^ over the 14‐day period. Mean energy availability for the injured participant was 24.0 kcal·kg^−1^·FFM^−1^ over the 14‐day period. There was no significant difference in energy availability for the non‐injured cohort across the pre‐season and one‐match microcycle (6.8 ± 7.9 kcal·kg^−1^·FFM^−1^; ES = 0.87; *p =* 0.125; Figure 4D).

## 4 Discussion

This study investigated the total, resting and activity energy expenditure, diet‐induced thermogenesis, water turnover, dietary intake and distribution, of senior male professional soccer players during pre‐season training with criterion methods. It was hypothesised that TEE would be greater during the microcycle including match play compared with the training‐only microcycle, and that dietary intake would not fully compensate for this increased energy requirement. However, the results did not support this hypothesis, as TEE remained similar between microcycles, likely due to compensatory adjustments in session duration and overall weekly load. Furthermore, only one player completed the full 90 min of match play, while three players completed 45 min, which may have attenuated the expected increase in TEE at group level. The training structure and intensity observed during the current study, comprising six training days per week, one to two daily sessions, and the introduction of friendly matches, were consistent with previous descriptions of pre‐season preparation in professional football (Anderson et al. 2022). Players did not achieve energy balance and failed to meet the minimum carbohydrate recommendations for pre‐season training. Carbohydrate intake was not periodised across rest, single‐training, double‐training, or match days, or between weeks, while protein intake met recommended targets. In contrast, fat intake was higher than current recommendations. Collectively, these findings provide novel evidence to support nutrition practitioners when coaching senior male players during the pre‐season mesocycle.

Senior male soccer players had energy requirements representative of a low‐active to active lifestyle during pre‐season training (PAL: 1.53–1.85 AU) (National Academies of Sciences E and M 2023). This is surprising, considering that players achieved high training loads across the assessment period (accumulated weekly duration: ∼560 min; distance: ∼40 km; HSR: ∼2200 m). Mean TEE are similar to in‐season values reported by DLW in the Eredivisie (13.8 ± 1.5 MJ⋅day^−1^; PAL 1.75 ± 0.13 AU; weekly duration ∼440 min; distance: ∼35 km) (Brinkmans et al. 2019), although appeared lower than those observed in the English Premier League (14.9 ± 2.4 MJ⋅day^−1^; weekly duration ∼320 min; distance: ∼27 km; HSR: ∼1300m) (Anderson, Orme, et al. 2017) and Japanese Professional Football League (14.8 ± 1.7 MJ⋅day^−1^; PAL 2.19 ± 0.31 AU) (Ebine et al. 2002). Despite the higher external loads observed in the present study compared with these in‐season cohorts, TEE remained similar, likely due to the absence of multiple competitive fixtures. There was no significant difference in the energy requirements of senior male soccer players during a pre‐season training and one‐match microcycle. In contrast to our hypothesis, the results indicate that player fuelling requirements are potentially repeatable during pre‐season microcycles that include training only, or a combination of training and match loads. Although match play was introduced in the one‐match microcycle, mean session duration was significantly shorter than the training‐only microcycle (89 vs. 107 min), while total distance covered remained similar between weeks. The number of double training days was also consistent between weeks, however the one‐match microcycle involved greater HSR, due to the inclusion of match play. This reflects a shift towards higher‐intensity, football‐specific preparation as the pre‐season progressed. Consequently, the energetic contribution of higher intensity activity in the one‐match microcycle likely offset the longer, lower intensity sessions performed in the training‐only microcycle, resulting in a comparable cumulative cost. Additionally, limited match exposure (four players x 45 min) further attenuated any additional increase in TEE. Meanwhile, participant 1 completed 118 min of match‐play during the one‐match microcycle, resulting in a modest increase in TEE of 1.11 MJ⋅day^−1^. This increase aligns with previous research reporting slightly greater mean TEE across two‐versus one‐match microcycles (∼+1 MJ⋅day^1^) (Anderson, Orme, et al. 2017; Ebine et al. 2002). Ultimately, such increases appear modest, with absolute TEE appearing to vary to a greater degree in relation to player FFM versus changes in overall load (Brinkmans et al. 2019).

We present criterion assessed TEE in a senior male professional soccer player immediately following injury. The injury led to a substantial reduction in weekly training load during the subsequent microcycle (duration: −402 min; distance: −28,041 m; HSR: −1052 m; sRPE: −1963 AU), representing an approximate 60% reduction in external load compared with the preceding week. This decrease in training load was accompanied by a proportional reduction in TEE of −5.31 MJ⋅day^−1^ (−34%), corresponding to a PAL decrease of −0.61 AU and AEE decrease of −5.37 MJ⋅day^−1^. These values are considerably lower than the TEE reported for an English Premier League male soccer player during anterior cruciate ligament rehabilitation (13.30 MJ⋅day^−1^) (Anderson et al. 2019). It is worth noting that the current case study was observed during the acute injury phase, rather than the return to participation phase (Ardern et al. 2016). As a result, more sessions were completed in the case study by Anderson and colleagues (eight resistance sessions, daily off‐feet conditioning, and hydrotherapy sessions vs. five resistance and two hydrotherapy sessions in this study) (Anderson et al. 2019). Clearly, further research is now required to establish the energy requirements of soccer players throughout distinct stages of the return to play pathway to enable more precise nutrition support during recovery from injury.

Senior male professional soccer players did not achieve daily energy and macronutrient recommendations for pre‐season training. In alignment with our hypothesis, mean carbohydrate intake (∼2.8 g⋅kg^−1^⋅day^−1^) was below the lower end of current UEFA recommendations for pre‐season training (4–8 g⋅kg^−1^⋅day^−1^), indicating sub‐optimal fuelling relative to football‐specific guidance (Collins et al. 2021). Mean intakes were also 3.2 and 5.2 g⋅kg^−1^⋅day^−1^ lower than the upper end of recommendations for single (6 g⋅kg^−1^⋅day^−1^) and double (8 g⋅kg^−1^⋅day^−1^) training days, respectively. Moreover, carbohydrate intake did not differ significantly across rest, single‐training, double‐training, or match day(s). This absence of dietary periodisation contrasts with reports from English Premier League players (Anderson, Orme, et al. 2017), who demonstrated significantly higher carbohydrate intakes on match days (6.4 ± 2.2 g⋅kg^−1^⋅day^−1^). Notably, no players in this study achieved intakes above 6 g⋅kg^−1^⋅day^−1^ on match day (−1 or +1). In contrast, participants consistently achieved dietary protein recommendations (1.6–2.2 g⋅kg^−1^⋅day^−1^), while mean fat intake (40% of TEI) exceeded football‐specific nutrition recommendations (20%–35% of total energy intake) (Collins et al. 2021; Thomas et al. 2016).

We provide evidence that senior male professional soccer players did not achieve macronutrient distribution targets at key time points during training and match days. Relative carbohydrate intakes at breakfast were 0.7 and 1.5 g⋅kg^−1^⋅day^−1^ lower than minimum recommendations for pre‐season single (1 g⋅kg^−1^⋅day^−1^) and double training days (2 g⋅kg^−1^⋅day^−1^), respectively (Anderson et al. 2022; Collins et al. 2021). Likewise, relative carbohydrate intakes post‐training (at lunch) were 0.8 and 1.3 g⋅kg^−1^⋅day^−1^ lower than recommended (single and double training day targets: 1.5–2.0 g⋅kg^−1^⋅day^−1^) (Anderson et al. 2022). Similar under‐fuelling of dietary carbohydrate has been demonstrated by senior male players from the English Premier League at breakfast (< 1.0 g⋅kg^−1^⋅day^−1^) and lunch (< 1.0 g⋅kg^−1^⋅day^−1^) on training days within a competitive two‐match microcycle (Anderson, Naughton, et al. 2017). Players did achieve carbohydrate (∼1 g⋅kg^−1^⋅day^−1^) and protein (0.5 ± 0.3 g⋅kg^−1^⋅day^−1^) recommendations for the first hour post‐competition (Collins et al. 2021; Thomas et al. 2016; Beelen et al. 2010) on match‐day, although failed to achieve carbohydrate recommendations over the following 3 hours. Collectively, these results suggest a lack of appropriate fuelling at key time points around pre‐season training and match‐play, which have important applied implications for practitioners when supporting players during the pre‐season mesocycle.

### 4.1 Practical Implications

Current guidelines for professional male soccer players suggest daily protein and fat intakes of 1.6–2.2 g⋅kg^−1^⋅day^−1^ and 20%–35% of total energy intake, respectively (Collins et al. 2021). This translates to 128–176 g⋅day^−1^ (2.14–2.95 MJ⋅day^−1^) of protein and 71–124 g⋅day^−1^ (2.68–4.67 MJ⋅day^−1^) of fat for a typical 80 kg male soccer player with 70 kg FFM. Based on the DLW assessed TEE presented in this study, this leaves 5.56–8.35 MJ⋅day^−1^ for dietary carbohydrate (or 4.2–6.2 g⋅kg^−1^⋅day^−1^; 332–498 g⋅day^−1^ for an 80 kg player). These carbohydrate recommendations sit within the lower to middle range for elite male soccer players across pre‐season (4–8 g⋅kg^−1^⋅day^−1^) (Collins et al. 2021), necessitating day‐to‐day carbohydrate periodisation. In practise, individual goals such as reducing fat mass or increasing lean mass during pre‐season may intentionally modify these targets, leading to short‐term energy deficits or macronutrient adjustments. Practitioners should therefore balance team‐based recommendations with individual body‐composition objectives. Moreover, practitioners should consider the dietary fat (and potentially alcohol) intake of players when prioritising carbohydrate intake, or when supporting player body composition. Meanwhile practitioners are encouraged to provide high carbohydrate meals and snack options that players look forward to consuming both before and after training/competition, challenging habitual under‐fuelling behaviour(s). Food items such as freshly made pancakes, crepes, French toast, and waffles may offer practical nutrition solutions at breakfast (pre‐training), while liquid options suit the increased thermal stress and reduced appetite typically experienced post‐training (recovery fruit smoothies, mocktails, and carbohydrate‐electrolyte drinks) or competition (decaffeinated fizzy drinks, and milkshakes) (Hulton et al. 2022).

### 4.2 Limitations and Strengths

This study utilised the DLW technique to measure TEE, which can introduce error at an individual level, despite reporting excellent validity at a group level (Speakman et al. 2021). Moreover, the DLW technique cannot typically provide accurate assessment of day‐to‐day variations in energy expenditure (Klein et al. 1984), which prevented assessment of TEE across single training, double training, match, or rest days. Dietary intake was assessed using a version of the RFPM, which, although practical and ecologically valid, is known to systematically underestimate total energy intake by approximately 10%–15%, and to show variable reliability, particularly when assessing complex meals. The coefficients of variation presented in Supporting Information S1: Figure 1, illustrate this inter‐individual variability. Several steps were taken to minimise potential error, including researcher assistance at all meal opportunities, an image‐based database of weighed standard portion sizes, and independent researcher dietary analysis. Nonetheless, some underestimation of intake relative to DLW‐assessed expenditure likely occurred. The estimation of EA in this study was calculated using energy intake minus AEE, which differs from the conventional calculation using energy intake minus EEE. Consequently, our EA values are not directly comparable with published thresholds and should be interpreted as contextual indicators of EA. Finally, the small sample of six players from one club may limit the generalisability of study findings to the wider population. Future research should aim to build on these limitations by investigating how energy requirements change on a day‐to‐day or session‐to‐session basis during the pre‐season mesocycle and during injury, while further work is now required to investigate preventative measures of inter‐researcher error displayed during dietary analysis in Supporting Information S1: Figure 1.

## 5 Conclusion

This study provides the first criterion‐assessed energy expenditure, water turnover and dietary intake data for senior male professional soccer players during pre‐season training. Total energy expenditure remained consistent across training‐only and training‐plus match microcycles, with mean values comparable to in‐season competition weeks. Despite high training loads, players did not adjust their dietary energy or carbohydrate intake according to daily or session demands and failed to achieve established nutritional recommendations, particularly before and after training and match play. Collectively, these findings reveal a persistent misalignment between energy expenditure and dietary intake during pre‐season and highlight the need for targeted, periodised nutrition strategies to optimise fuelling, recovery and adaptation in elite football.

## Funding

The authors have nothing to report.

## Conflicts of Interest

The authors declare no conflicts of interest.

## Supporting information


Supporting Information S1
