# Supplementary material for: Pre‐Season Total Energy Expenditure and Dietary Intake of Professional Male Soccer Players: A Doubly Labelled Water Study
Source: Eur J Sport Sci. 2026 Feb 27;26(3):e70149. doi: 10.1002/ejsc.70149 (PMC12948648; doi:10.1002/ejsc.70149)
Supplement: Supplementary file 1 — Supporting Information S1 [file EJSC-26-e70149-s001.docx]

**Supplementary Material: Pre-season total energy expenditure and dietary intake of professional male soccer players: a doubly labelled water study**

**Supplementary Table 1:** GPS-derived external and internal load across weekly microcycles. Values are mean ± SD.

| **Player**  **(Position)** | **Duration**  **(min)** | | **Total Distance**  **(m)** | | **HSR + Sprint Distance**  **(m)** | | | **sRPE**  **(AU)** | |
| --- | --- | --- | --- | --- | --- | --- | --- | --- | --- |
| *Microcycle* | *Pre-season* | *Match* | *Pre-season* | *Match* | | *Pre-season* | *Match* | *Pre-season* | *Match* |
| 1 (CM) | 132 ± 71 | 118 ± 30 | 8671 ± 3028 | 8860 ± 1797 | | 535 ± 355 | 581 ± 226 | 662 ± 344 | 574 ± 135 |
| 2 (CD) | 107 ± 59 | 81 ± 38 | 6961 ± 2007 | 6259 ± 1995 | | 205 ± 153 | 346 ± 257 | 539 ± 282 | 361 ± 173 |
| 3 (CM) | 107 ± 58 | 94 ± 25 | 7382 ± 2502 | 7149 ± 1170 | | 368 ± 394 | 477 ± 284 | 521 ± 240 | 430 ± 115 |
| 4 (F) | 81 ± 40 | 64 ± 33 | 5693 ± 2249 | 5936 ± 1929 | | 235 ± 173 | 445 ± 49 | 427 ± 281 | 363 ± 198 |
| 6 (WD) | 108 ± 60 | 81 ± 47 | 7896 ± 2596 | 6445 ± 3795 | | 421 ± 420 | 492 ± 323 | 532 ± 241 | 410 ± 244 |

HSR = High speed running (>19.8 km/h); sRPE = session rating of perceived exertion. CM = central midfielder; CD = central defender; F = forward; GK = goalkeeper; WD = wide defender.

**Supplementary Table 2:** Dietary intake across weekly microcycles. Values are mean ± SD.

| **Player**  **(Position)** | **Energy**  **(MJ·day^-1^**  **kcal·day^-1^)** | | **Protein**  **(g·day^-1^**  **g·kg·day^-1^)** | | **Carbohydrate**  **(g·day^-1^**  **g·kg·day^-1^)** | | **Fat**  **(g·day^-1^**  **g·kg·day^-1^)** | | **Alcohol**  **(g·day^-1^**  **g·kg·day^-1^)** | |
| --- | --- | --- | --- | --- | --- | --- | --- | --- | --- | --- |
| *Microcycle* | *Pre-season* | *Match* | *Pre-season* | *Match* | *Pre-season* | *Match* | *Pre-season* | *Match* | *Pre-season* | *Match* |
| 1 (CM) | 2667 ± 501 | 2694 ± 422 | 186 ± 36  3.0 ± 0.6 | 157 ± 26  2.5 ± 0.4 | 220 ± 61  3.5 ± 1.0 | 234 ± 77  3.8 ± 1.2 | 116 ± 35  1.9 ± 0.6 | 123 ± 21  2.0 ± 0.3 | 0 ± 0  0.0 ± 0.0 | 3 ± 9  0.1 ± 0.1 |
| 2 (CD) | 2664 ± 599 | 2647 ± 507 | 162 ± 50  2.0 ± 0.6 | 154 ± 27  1.9 ± 0.3 | 228 ± 49  2.8 ± 0.6 | 226 ± 51  2.7 ± 0.6 | 122 ± 38  1.5 ± 0.5 | 125 ± 36  1.5 ± 0.4 | 0 ± 0  0.0 ± 0.0 | 0 ± 0  0.0 ± 0.0 |
| 3 (CM) | 2429 ± 395 | 2665 ± 639 | 171 ± 28  2.3 ± 0.4 | 159 ± 40  2.1 ± 0.5 | 175 ± 41  2.4 ± 0.6 | 225 ± 91  3.0 ± 1.2 | 115 ± 30  1.5 ± 0.4 | 125 ± 31  1.7 ± 0.4 | 0 ± 0  0.0 ± 0.0 | 0 ± 0  0.0 ± 0.0 |
| 4 (F) | 3135 ± 537 | 2920 ± 466 | 201 ± 33  2.4 ± 0.4 | 160 ± 38  1.9 ± 0.5 | 227 ± 61  2.7 ± 0.7 | 242 ± 54  2.9 ± 0.6 | 154 ± 33  1.8 ± 0.4 | 141 ± 28  1.7 ± 0.3 | 2 ± 5  0.0 ± 0.1 | 6 ± 11  0.1 ± 0.1 |
| 5 (GK) | 1939 ± 442 | 2120 ± 580 | 150 ± 28  1.8 ± 0.3 | 164 ± 35  1.9 ± 0.4 | 145 ± 43  1.7 ± 0.5 | 184 ± 78  2.2 ± 0.9 | 85 ± 27  1.0 ± 0.3 | 80 ± 27  0.9 ± 0.3 | 0 ± 0  0 ± 0 | 3 ± 5  0.0 ± 0.1 |
| 6 (WD) | 2235 ± 419 | 2387 ± 687 | 148 ± 41  1.9 ± 0.5 | 154 ± 55  1.9 ± 0.7 | 187 ± 32  2.4 ± 0.4 | 206 ± 89  2.6 ± 1.1 | 99 ± 26  1.2 ± 0.3 | 92 ± 32  1.2 ± 0.4 | 0 ± 0  0.0 ± 0.0 | 17 ± 31  0.2 ± 0.4 |

CM = central midfielder; CD = central defender; F = forward; GK = goalkeeper; WD = wide defender.

**Supplementary Table 3:** Energy expenditure and water turnover across weekly microcycles. Values are mean ± SD.

| **Player (Position)** | **TEE**  **(MJ·day^-1^**  **kcal·day^-1^)** | | **REE**  **(MJ·day^-1^**  **kcal·day^-1^)** | **AEE**  **(MJ·day^-1^**  **kcal·day^-1^)** | | **DIT**  **(MJ·day^-1^**  **kcal·day^-1^)** | | **WT**  **(L·day^-1^**  **ml·kg·FFM^-1^)** | |
| --- | --- | --- | --- | --- | --- | --- | --- | --- | --- |
| *Microcycle* | *Pre-season* | *Match* | *Pre-season* | *Pre-season* | *Match* | *Pre-season* | *Match* | *Pre-season* | *Match* |
| 1 (CM) | 11.97  2862 | 13.08  3126 | 7.49  1790 | 3.37  805 | 4.46  1066 | 1.12  267 | 1.13  269 | 4.8  93.7 | 4.8  94.8 |
| 2 (CD) | 17.00  4063 | 12.72  3040 | 8.38  2002 | 7.51  1794 | 3.23  773 | 1.11  266 | 1.11  265 | 4.8  70.0 | 4.3  62.1 |
| 3 (CM) | 13.86  3313 | 11.58  2769 | 6.83  1632 | 6.02  1438 | 3.64  870 | 1.02  243 | 1.12  267 | 5.3  82.0 | 5.2  80.1 |
| 4 (F) | 15.70  3752 | 14.33  3425 | 7.95  1900 | 6.43  1537 | 5.16  1233 | 1.32  316 | 1.22  292 | 6.7  88.6 | 5.8  77.5 |
| 5 (GK) | 15.31  3658 | 12.70  3036 | 9.18  2193 | 5.32  1271 | 2.64  631 | 0.81  194 | 0.89  213 | 5.2  75.2 | 4.7  67.2 |
| 6 (WD) | 15.70  3752 | 10.39  2484 | 8.68  2074 | 6.09  1455 | 0.72  171 | 0.94  224 | 1.00  239 | 5.6  86.9 | 5.1  78.6 |

TEE = Total energy expenditure; REE = resting energy expenditure; AEE = activity energy expenditure; DIT = diet-induced thermogenesis.; WT = water turnover.

| **Player**  **(Position)** | **Time Sedentary**  **(% of total wear time)** | | **Time Light**  **(% of total wear time)** | | **Step count** | |
| --- | --- | --- | --- | --- | --- | --- |
| *Microcycle* | *Pre-season* | *Match* | *Pre-season* | *Match* | *Pre-season* | *Match* |
| 1 (CM) | 72 ± 6 | 72 ± 3 | 28 ± 6 | 28 ± 3 | 10,347 ±  2,097 | 11,091 ±  1,233 |
| 2 (CD) | 74 ± 3 | 74 ± 4 | 26 ± 3 | 26 ± 4 | 9131 ±  1162 | 9499 ±  1879 |
| 3 (CM) | 73 ± 4 | 69 ± 4 | 27 ± 4 | 31 ± 4 | 9929 ±  2660 | 11,801 ±  2,628 |
| 4 (F) | 71 ± 3 | 74 ± 3 | 29 ± 3 | 26 ± 3 | 10,922 ±  1,140 | 10,447 ±  874 |
| 5 (GK) | 70 ± 9 | 72 ± 5 | 30 ± 9 | 28 ± 5 | 11,246 ±  2,310 | 12,593 ±  3,438 |
| 6 (WD) | 74 ± 2 | 73 ± 6 | 26 ± 2 | 27 ± 6 | 9232 ±  934 | 10,571 ±  2,214 |

**Supplementary Table 4:** Individual accelerometer-derived physical activity data across weekly microcycles. Values are mean ± SD.

CM = central midfielder; CD = central defender; F = forward; GK = goalkeeper; WD = wide defender.

**
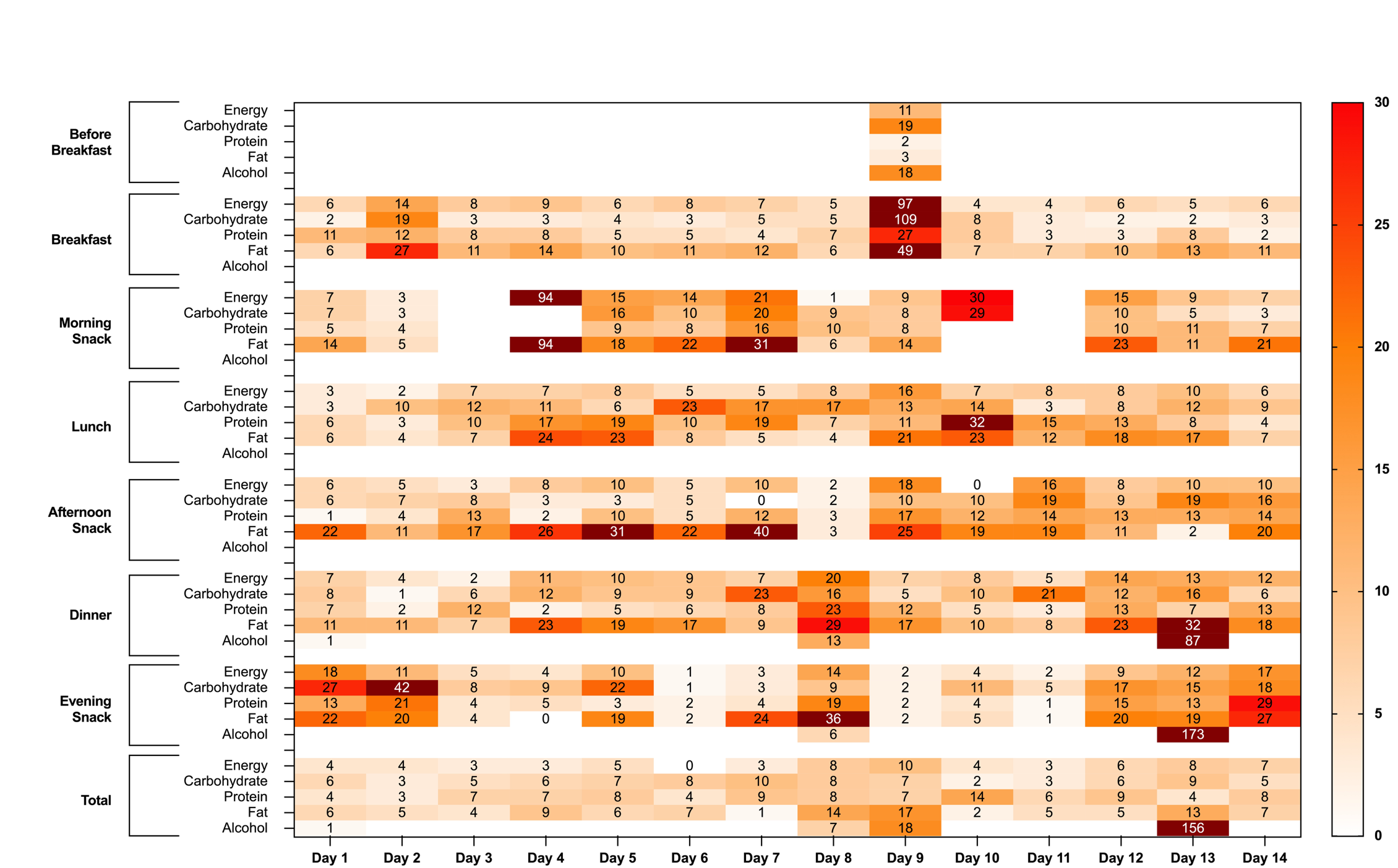
**

**Supplementary Figure 1:** Inter-researcher coefficient of variation (%) for dietary energy and macronutrient intake across all days, eating occasions, and participants.
